# Ginsenoside Rb1 Modulates the Migration of Bone-Derived Mesenchymal Stem Cells through the SDF-1/CXCR4 Axis and PI3K/Akt Pathway

**DOI:** 10.1155/2022/5196682

**Published:** 2022-03-10

**Authors:** Yimei Liu, Ninghua Liu, Xiangyang Li, Zhe Luo, Jing Zhang

**Affiliations:** ^1^Department of Critical Care Medicine, Zhongshan Hospital Fudan University, Shanghai 200032, China; ^2^Department of Facial Plastic and Reconstructive Surgery, Eye & ENT Hospital of Fudan University, Shanghai 200031, China; ^3^Department of Nursing, Zhongshan Hospital of Fudan University, Shanghai 200032, China

## Abstract

**Methods:**

Wound-healing assay and Transwell assay were utilized to evaluate the effect of ginsenoside Rb1 on the migration of BMSCs. RT-PCR and Western blotting were performed to evaluate the expression of stromal-derived factor 1 (SDF-1), C-X-C chemokine receptor type 4 (CXCR4), phosphatidylinositol 3-kinase (PI3K), and protein kinase B (PKB; AKT).

**Results:**

Ginsenoside Rb1 significantly enhanced the migration of BMSCs through the activation of SDF-1, CXCR4, p-PI3K/PI3K, and p-Akt/Akt relative expression. Furthermore, this stimulus was blocked by the pretreatment with AMD3100 and LY294002.

**Conclusions:**

Ginsenoside Rb1 facilitated the migration of BMSCs through the activation of the SDF-1/CXCR4 axis and PI3K/Akt pathway.

## 1. Introduction

The skin is the first barrier of the body against the external environment. When the skin is injured, the wound-healing process is required to restore the skin barrier. Wound healing is a conserved evolutionary process that involves angiogenesis, inflammation response, new tissue formation, and tissue remodeling [1, 2]. During skin homeostasis and repair, stem cells located in the skin provide epidermal self-renewal to maintain the repair process [3]. Mesenchymal stem cells (MSCs) have the ability to self-renew and differentiate into a variety of tissue-forming cell lineages and secrete various bioactive factors to support all events during the skin regeneration process including inflammation, angiogenesis, granulation tissue formation, cell migration, and reepithelialization [4]. Several studies have shown that the cell proliferation and migration capacity of stem cells were impaired under extensive oxidative stress, and antioxidative agents may exert a protective effect to enhance the proliferation and migration of stem cells [5–7].

Panax ginseng is the most commonly used traditional herbal medicine in Asia over the past 2000 years and utilized as active components in the skin, cardiovascular, endocrine, immune, and central nervous systems [8, 9]. Ginsenoside Rb1 is the major bioactive component in ginseng. Many studies demonstrated that ginsenoside Rb1 protected endothelial cells from oxidative stress-induced cell aging [10] and improved intestinal epithelial wound healing [11]. Another study indicated that ginsenoside Rb1 promoted the proliferation of hair follicles and dermal papilla cells [12]. These studies implied that ginsenoside Rb1 may have therapeutic effects in cutaneous wound healing.

The SDF-1/CXCR4 axis was identified to play a key role in the mobilization and migration of MSCs in a rat model of a bone defect [13]. On the other hand, SDF-1/CXCR4 was proved to regulate the migration of MSCs to the damaged area after traumatic brain injury in rats [14]. Li et al. [15] showed that the SDF-1/CXCR4 axis facilitated dental pulp stem cell migration through the PI3K/Akt pathways, which is involved in a variety of cellular processes such as cell proliferation and migration, apoptosis, invasion, angiogenesis, and metastasis [16, 17]. We previously reported that ginsenoside Rb1 protected mice against acute liver injury by attenuating oxidative stress and the inflammatory response [18]. However, the effect and the underlying mechanism of ginsenoside Rb1 on MSCs are largely unknown. Clarifying these aspects of ginsenoside Rb1 could illuminate its role in the wound-healing process.

## 2. Materials and Methods

### 2.1. Ethical Approval of Study Protocol

Mice (C57BL/6, 8-10 weeks) were purchased from the laboratory animal resources of the Chinese Academy of Sciences and raised in the animal laboratory of Fudan University. The research protocol has been approved by the Ethics Committee of Zhongshan Hospital of Fudan University. All of our experiments were conducted according to relevant guidelines and regulations.

### 2.2. Reagents

Ginsenoside Rb1 (purity of about 98%) was purchased from Meilun Pharmaceuticals Inc. (Dalian, China). The CD29, CD34, CD90, CD105, and relevant isotype antibodies for flow cytometry analysis were purchased from BD Biosciences, UK. The antibodies of SDF-1, CXCR4, p-PI3K, PI3K, p-AKT, AKT, and GAPDH used for the Western blot analysis were purchased from Cell Signaling Technology, USA. For the pretreatment of AMD3100 (CXCR4 antagonist, A5602, Sigma) and LY294002 (PI3K inhibitor, L9908, Sigma), the BMSCs were cocultured with AMD3100 and LY294002 for 1 hour at 37°C prior to the administration of ginsenoside Rb1.

### 2.3. Isolation and Multilineage Differentiation of BMSCs

Mice were anesthetized by intraperitoneal injection of chloral hydrate (300 mg/kg body weight) and then euthanized. The femur and tibia were removed and transferred into a sterile plate. The ends of the femur were removed, 3 mL of phosphate-buffered saline (PBS) was used to flush out the bone marrow, and a cell suspension was collected and centrifuged at 1000 rpm for 10 min. Cells were dispensed into tissue culture flasks (Corning Glass Works, Corning, NY) containing 5 mL complete medium composed of DMEM, 10% FBS, 100 U/mL penicillin, and 100 g/mL streptomycin (Gibco). After 24 h, the medium was changed, and nonadherent cells were discarded. The cultured cells were observed with a microscope, and their expansion and cell shape were observed. To evaluate the multilineage differentiation capacity of the isolated BMSCs, BMSCs from the 3^rd^ passage were cultured with adipogenic, chondrogenic, and osteogenic inductive media. After 2-3 weeks of induction, differentiation of BMSCs was detected by relevant histological methods.

### 2.4. Flow Cytometry Analysis

The cultured BMSCs were treated with trypsin-EDTA and then subjected to centrifugation at 300 g for 5 minutes. BMSCs were transferred into tubes at 0.5 × 10^6^ cells and incubated with the antibodies. At the end of the incubation period, BMSCs were washed with PBS and centrifuged for 5 min at 300 g. The fluorescence intensities were measured by flow cytometer (Merck Millipore, UK), and data were calculated using the FlowJo software (Tree Star Inc., Oregon).

### 2.5. Wound-Healing Assay

BMSCs at passages 4 to 6 were used in subsequent experiments. BMSCs were transferred into a six-well plate at a density of 1.5 × 10^6^ cells/mL. After 24 h, the cell confluence reached 100%. Then the cellular monolayer on the plates was scraped with a pipette tip of 200 *μ*L to make a manual “wound,” and the baseline was recorded. The floating cell fragments were removed with PBS buffer. After incubation for 24 h in a complete medium, the migration of BMSCs was recorded. Then five randomly selected areas of the same size were quantified with the ImageJ software (NIH, Bethesda, MD, USA). Experiments were repeated at least three times, and an independent photograph was selected as the representation.

### 2.6. Transwell Migration Assay

The Transwell migration assay was performed by inserting a Transwell polyester membrane filter with 8 *μ*m pores polycarbonate membrane in 24-well culture plates at a density of 0.5 × 10^6^ cells/mL per Transwell (upper chamber). Various coculture conditions were used in the bottom chamber of Transwell. After 12 hours of incubation, the cells in the upper part of the chamber were removed, and the cells on the membranes of the Transwell insert were stained with 0.2% crystal violet (Beyotime, Haimen, China). The number of cells was counted with a light microscope (Olympus, Tokyo, Japan).

### 2.7. Real-Time RT-PCR

The whole RNA in each group was isolated with TRIzol reagent and followed reverse transcription into cDNA based on the manufacturer's instructions of the TaKaRa kit (TaKaRa Biotechnology, Shiga, Japan). The RT-PCR experiment was established with the QuantiTect SYBR Green RT-PCR Kit commercial from Thermo Scientific. The PCR experiment condition was as follows: 10 min at 95°C and then followed 40 cycles at changing temperatures/times (95°C, 60°C, and 72°C for 20 s, respectively). The melting curve analysis was analyzed at the final time of the amplification time. The gene differences of the two groups were collected by normalizing the Ct values to *β*-actin. The initial sequences were as follows: mouse SDF-1 sense primer: 5′-GAG AGC CACATC GCC AGA GC-3′ and mouse SDF-1 antisense primer: 5′-GGA TCC ACT TTA ATT TCG GGT CAA-3′; mouse CXCR4 sense primer: 5′-GAC CGC CTT TAC CCC GAT AGC and mouse CXCR4 antisense primer: 5′-ACC CCC AAA AGG ATG AAG GAG TC-3′; and mouse GAPDH sense primer: 5′-GTT ACC AGG GCT GCC TTC TC-3′ and mouse GAPDH antisense primer: 5′-GAT GGT GAT GGG TTT CCC GT-3′.

### 2.8. Western Blotting

The protein was extracted, and protein concentrations were measured with a BCA kit (Beyotime, Shanghai, China) according to the manufacturer's instructions. Equal amounts of proteins were separated via SDS-PAGE and then transferred to a PVDF membrane. The membrane was blocked with a solution which contained 5% nonfat dry milk and PBS buffer overnight at 4°C. After blocking the nonspecific binding, the membrane was incubated with first antibodies for about 2.5 h at room temperature. The primary antibodies used in our study included anti-SDF-1 (1 : 1000; Abcam), anti-CXCR4 (1 : 1000; Abcam), anti-p-PI3K (1 : 1000; Cell Signaling Technology), anti-PI3K (1 : 1000; Cell Signaling Technology), anti-p-Akt (1 : 1000; Cell Signaling Technology), anti-Akt (1 : 1000; Cell Signaling Technology), and anti-GAPDH (1 : 1000; Abcam). Afterwards, the membranes were incubated with secondary antibodies with a ratio of 1 : 3000 for 1.5 h at room temperature. The immunoreactive bands were analyzed using an ECL detection kit. The Western blot band densitometry analysis was performed, and the data were analyzed with the ImageJ software.

### 2.9. Statistical Analyses

The data are shown in the form of the mean ± standard deviation (SD). Statistically significant differences between data (*p* < 0.05) were assessed by one-way analysis of variance (ANOVA) followed by post hoc Tukey's test or Student's *t*-test (SPSS version 17.0; SPSS Inc., Chicago, IL, USA).

## 3. Results

### 3.1. Morphology, Multilineage Differentiation and Surface Molecules Expression of BMSCs

**Figure 1:**
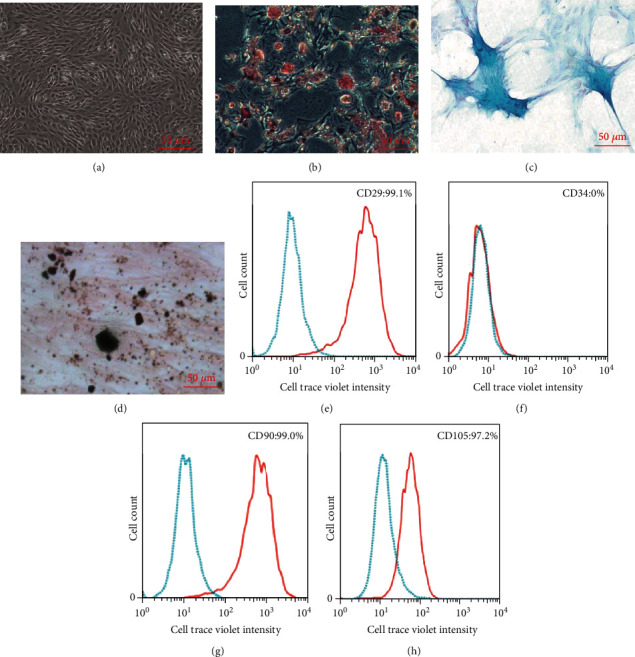
The morphology, multilineage differentiation, and surface molecules expression of BMSCs. (a) BMSCs of passage 1: cells tended to be uniform with typical fibroblast-like morphology. (b) BMSCs were incubated with adipogenic induction medium, and the cells were positively stained by Oil Red O. (c) BMSCs were incubated with chondrogenic induction medium, and the samples were positively stained by Alcian blue. (d) BMSCs were incubated with an osteogenic induction medium, and calcium deposits were noted by Von Kossa. (e–h) The surface molecules expression of BMSCs showed expression of CD29, CD90, and CD105 whilst lacking CD34 (red solid line). The isotype antibodies were used as negative controls (blue dotted line).

After the initial isolation and expansion, BMSCs of passage 1 tended to be uniform and show a stronger proliferative capacity into a monolayer with typical fibroblast-like morphology (Figure 1(a)). When BMSCs were incubated with adipogenic induction medium for 2 weeks, the cell morphology changed significantly from a spindle-shaped to intumescent morphology, and the cells were filled with adipose drops that were positively stained for Oil Red O (Figure 1(b)). After incubation in a chondrogenic induction medium for 3 weeks, the samples were positively stained by Alcian blue (Figure 1(c)). Calcium deposits were observed after 3 weeks of incubation with an osteogenic induction medium (Figure 1(d)); these deposits are biochemical features of bone mineralization. Phenotypic identification was analyzed by flow cytometry, the results showed that MSC-specific markers CD29 was positive with a mean ± SD of 99.1 ± 0.8%, CD90 was positive with a mean ± SD of 99.0 ± 0.6%, CD105 was positive with a mean ± SD of 97.2 ± 0.8%, all samples were negative for CD34, and these results demonstrated the high purity of BMSCs and the absence of hematopoietic contaminants [19].

### 3.2. Ginsenoside Rb1 Enhanced the Migration of BMSCs

**Figure 2:**
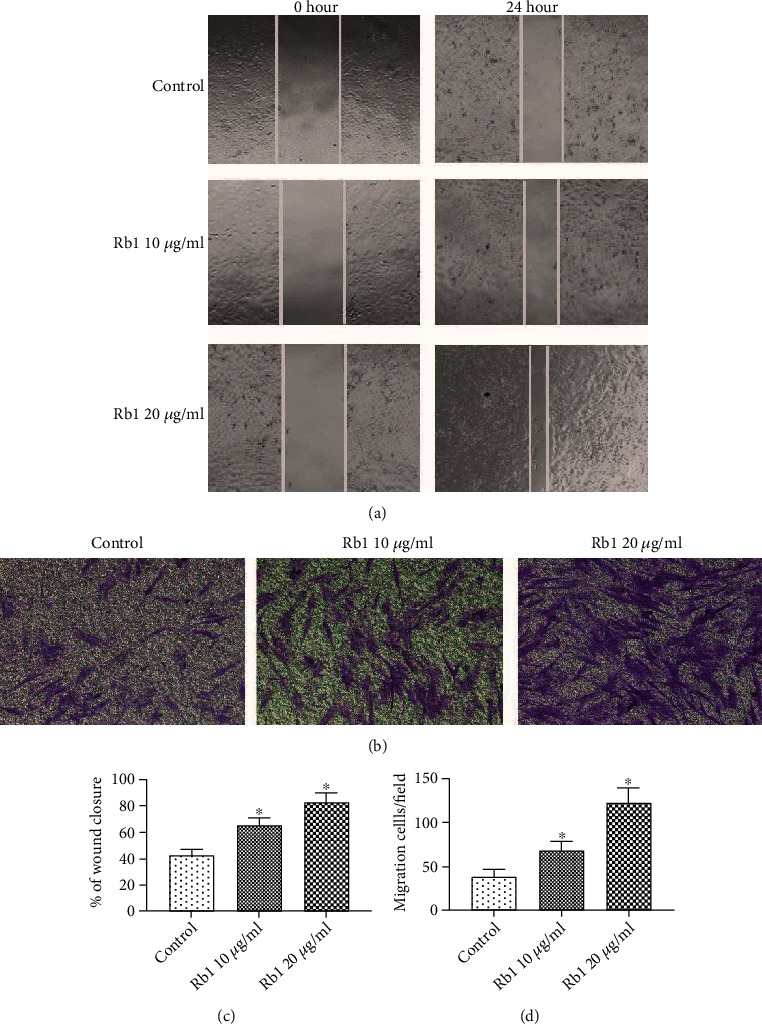
Ginsenoside Rb1 facilitated the migration of BMSCs. The BMSCs were incubated with different concentrations of ginsenoside Rb1 (0, 10, and 20 *μ*g/mL, respectively). (a) Representative images of the wound-healing assay in different groups, and wound sites were observed and photographed at 0 and 24 h (×200 original magnification). (b) Representative images of the Transwell assay in different groups, and cells migrated from the upper chamber to the bottom surfaces were stained with crystal violet and observed under a microscope (×200 original magnification). (c) Quantitative results of the wound-healing assay. (d) Quantitative results of the Transwell assay. Tests were performed at least three times. Data are shown as mean ± standard error of the mean. ^∗^*p* < 0.05 vs. the control group.

To evaluate the effect of ginsenoside Rb1 on the migration of BMSCs, we performed a wound-healing assay and Transwell assay. As shown in Figures 2(a) and 2(c), the wound coverage area was significantly increased in the Rb1 group compared with the control group (*p* < 0.05), and the wound closure rate was increased by approximately 100% upon exposure to Rb1 (20 *μ*g/mL) compared with that in the control group. Consistent with the data obtained in the wound-healing assay, the Transwell assay revealed that the number of migrated BMSCs in the lower chamber was significantly increased in the Rb1 group compared with that in the control group (Figures 2(b) and 2(d); ^∗^*p* < 0.05). These results indicated that ginsenoside Rb1 promoted the migration of BMSCs.

### 3.3. Ginsenoside Rb1 Upregulated the SDF-1/CXCR4 Axis and PI3K/Akt Pathway

**Figure 3:**
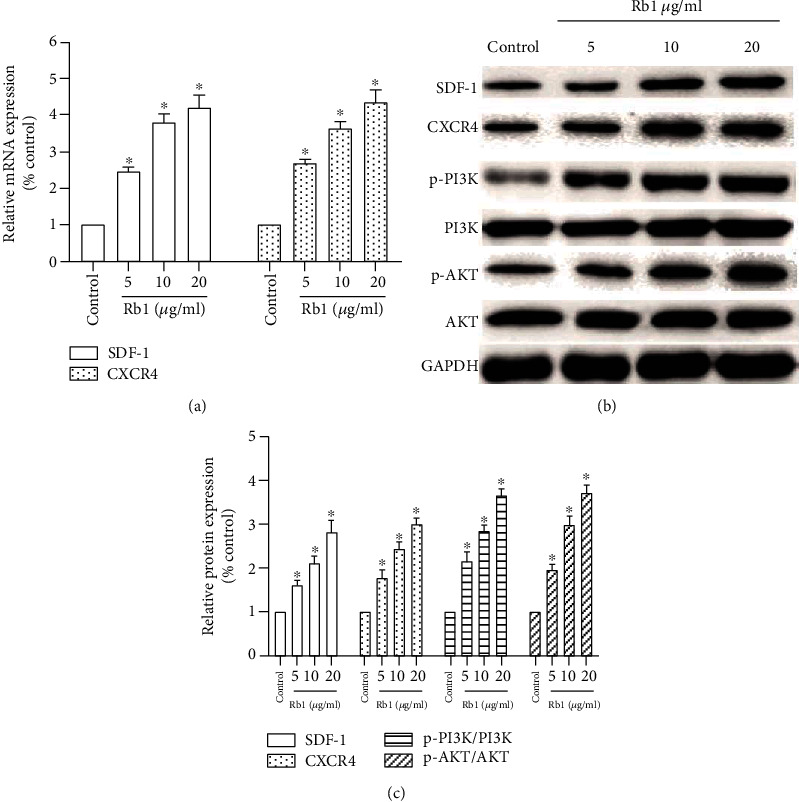
Effect of ginsenoside Rb1 on the SDF-1/CXCR4 axis and PI3K/Akt pathway. (a) The mRNA expressions of SDF-1 and CXCR4. (b) Western blot analysis of SDF-1, CXCR4, p-PI3K/PI3K, and p-Akt/Akt. (c) The relative protein content was quantified by densitometry. Results were performed at least 3 times. Data are shown as the means ± standard error of the mean. ^∗^*p* < 0.05 vs. the control group.

The SDF-1/CXCR4 axis and PI3K/Akt pathway played an important part in determining the migration ability of BMSCs. The effects of ginsenoside Rb1 on SDF-1/CXCR4 were investigated using RT-PCR. As shown in Figure 3(a), the mRNA expressions of SDF-1 and CXCR4 were significantly increased in the Rb1 group compared with that in the control group (*p* < 0.05). We next determined the protein expression of SDF-1, CXCR4, and PI3K/Akt by Western blot analysis. The results showed that ginsenoside Rb1 significantly increased the expression of SDF-1, CXCR4, p-PI3K/PI3K, and p-Akt/Akt relative expression (Figures 3(b) and 3(c); ^∗^*p* < 0.05). These results indicated that ginsenoside Rb1 significantly increased the expression of the SDF-1/CXCR4 axis and PI3K/Akt pathway.

### 3.4. CXCR4 Antagonist Suppressed the Migration of BMSCs and the Expression of the SDF-1/CXCR4 Axis and PI3K/Akt Pathway

**Figure 4:**
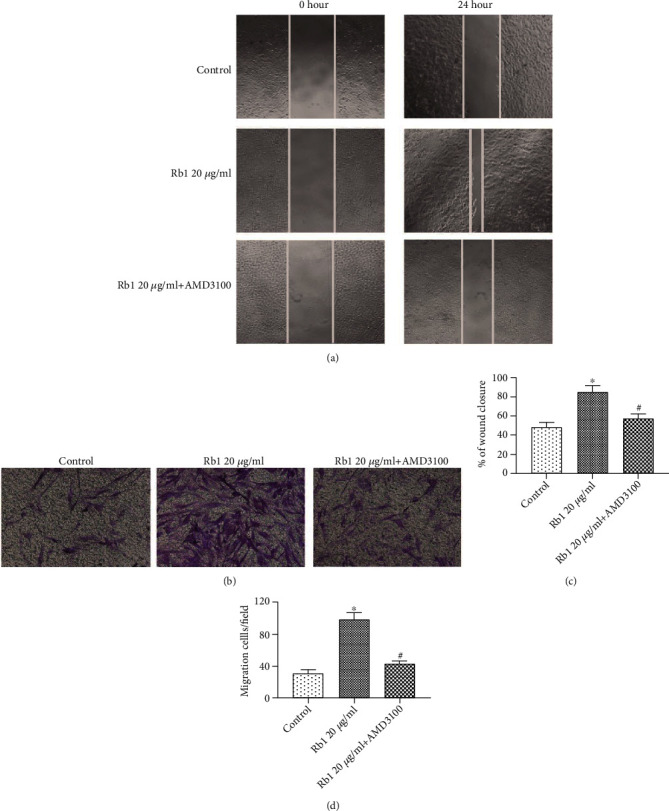
CXCR4 antagonist inhibited the migration of BMSCs. BMSCs were pretreated with AMD3100 for 1 hour at 37°C prior to the administration of ginsenoside Rb1. (a) Representative images of the wound-healing assay in different groups, and wound sites were observed and photographed at 0 and 24 h (×200 original magnification). (b) Representative images of the Transwell assay in different groups, and cells migrated from the upper chamber to the bottom surfaces were stained with crystal violet and observed under a microscope (×200 original magnification). (c) Quantitative results of the wound-healing assay. (d) Quantitative results of the Transwell assay. Tests were performed at least three times. Data are shown as mean ± standard error of the mean. ^∗^*p* < 0.05 vs. the control group. ^#^*p* < 0.05 versus the Rb1 20 *μ*g/mL group.

**Figure 5:**
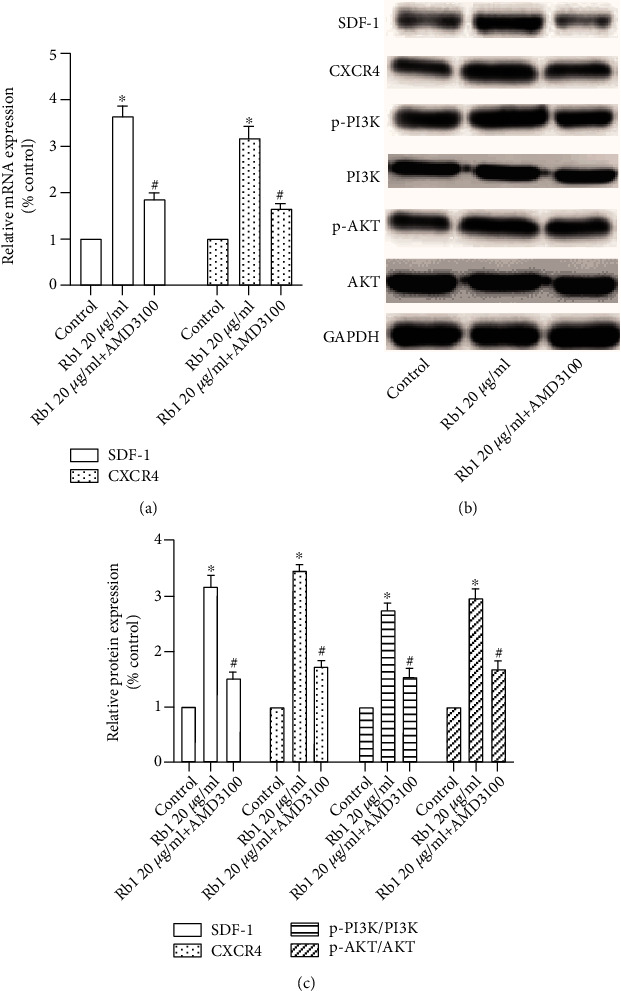
CXCR4 antagonist inhibited the activation of the SDF-1/CXCR4 axis and PI3K/Akt pathway. (a) The mRNA expressions of SDF-1 and CXCR4. (b) Western blot analysis of SDF-1, CXCR4, p-PI3K/PI3K, and p-Akt/Akt. (c) The relative protein content was quantified by densitometry. Results were performed at least 3 times. Data are shown as the means ± standard error of the mean. ^∗^*p* < 0.05 vs. the control group. ^#^*p* < 0.05 versus the Rb1 20 *μ*g/mL group.

To further clarify the molecular mechanism through which ginsenoside Rb1 affected the migration ability of BMSCs, AMD3100 (CXCR4 antagonist) was used prior to the administration of ginsenoside Rb1. Following pretreatment with AMD3100 for 1 h, the wound coverage and migrated cell numbers were significantly decreased in the Rb1+AMD3100 group compared with that in the Rb1 group (Figure 4; ^#^*p* < 0.05). The gene and protein expression of SDF-1 and CXCR4 were significantly decreased in the presence of AMD3100 (Figure 5; ^#^*p* < 0.05). Moreover, inhibitor studies revealed that inhibition of CXCR4 attenuated the relative expression of p-PI3K/PI3K and p-Akt/Akt, indicating that the SDF-1/CXCR4 axis may regulate the PI3K/Akt pathway in the Rb1-mediated increase in BMSC migration.

### 3.5. PI3K Inhibitor Suppressed the Migration of BMSCs Promoted by Ginsenoside Rb1

**Figure 6:**
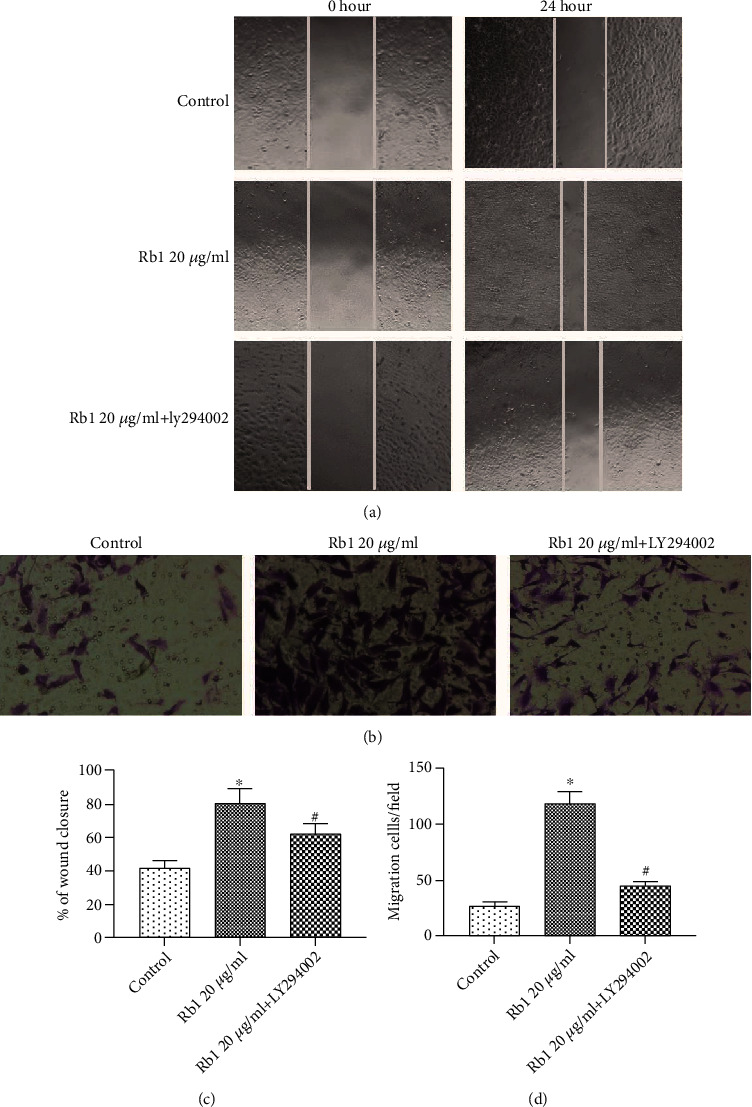
The PI3K inhibitor suppressed the migration process of BMSCs. BMSCs were pretreated with LY294002 for 1 hour at 37°C prior to the administration of ginsenoside Rb1. (a) Representative images of the wound-healing assay in different groups, and wound sites were observed and photographed at 0 and 24 h (×200 original magnification). (b) Representative images of the Transwell assay in different groups, and cells migrated from the upper chamber to the bottom surfaces were stained with crystal violet and observed under a microscope (×200 original magnification). (c) Quantitative results of the wound-healing assay. (d) Quantitative results of the Transwell assay. Tests were performed at least three times. Data are shown as mean ± standard error of the mean. ^∗^*p* < 0.05 vs. the control group. ^#^*p* < 0.05 versus the Rb1 20 *μ*g/mL group.

To determine the effect of the PI3K/Akt signaling pathway in the migration process of BMSCs, BMSCs were pretreated with or without LY294002, a PI3K inhibitor. We found that pretreatment of LY294002 reversed the increased migration promoted by ginsenoside Rb1, and the relative expressions of p-PI3K/PI3K and p-Akt/Akt were also downregulated as shown in Figure 6. These data demonstrated that ginsenoside Rb1 accelerated the migration process of BMSCs via the activation of the PI3K/Akt pathway.

## 4. Discussion

A cutaneous wound-healing process is highly regulated by several cellular processes, including hemostasis, inflammatory, proliferative, and maturation phases. The resident MSCs in various tissues are wildly involved in wound healing. The aim of stem cell therapy is to explore the function of these cells to improve tissue dysfunction and immune deficiency. However, the migration of MSCs was downregulated under high-glucose conditions [20], and this impaired migration ability may be related to the delayed wound healing in diabetes-induced wounds. Therefore, exploring a feasible and effective approach to enhance the migration ability of MSCs is of great clinical significance and will contribute to the development of therapies for wound healing. In our study, the 3^rd^ passage of BMSCs was cultured with adipogenic, chondrogenic, and osteogenic inductive media to evaluate the multilineage differentiation capability of BMSCs. Moreover, the surface molecular expression of BMSCs was analyzed by flow cytometry, the results showed that MSC-specific markers CD29, CD90, and CD105 were positive, all samples were negative for CD34 (Figure 1), and these results verified the identification of BMSCs.

Several different ginsenosides have been proved to promote the wound-healing process, such as ginsenosides Rg1, Rd, and Rb1 [21–23]. However, ginsenoside Rb1 may be the most potential candidate to increase the wound-healing process in diabetes-induced wounds. Ginsenoside Rb1 is one of the most active ingredients in ginseng and has multiple biological activities such as antidiabetic, antioxidative, and immunomodulating activity and cellular growth regulation. Many researchers focused on the effect of ginsenoside Rb1 on cellular and molecular healing processes. Shin et al. [24] found that ginsenoside Rb1 stimulated keratinocyte migration through the sphingosine-1-phosphate receptor-mediated pathway. Hou and Kim [25] indicated that ginsenoside Rb1 facilitated keratinocyte scratch wound healing by regulating senescent skin dermal fibroblasts. However, there have been few studies on the effect of ginsenoside Rb1 on BMSC migration. In this study, cell migration ability was evaluated by the wound-healing assay and Transwell migration assay, and the scratch coverage and migrated cell number were significantly increased in the ginsenoside Rb1 group, which suggested that ginsenoside Rb1 facilitated BMSC migration (Figure 2).

**Figure 7:**
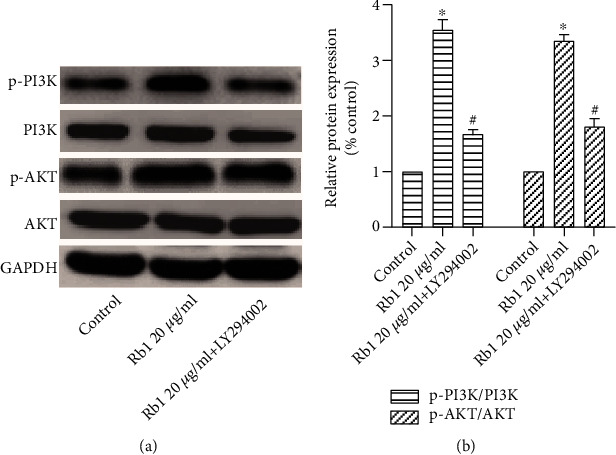
The PI3K inhibitor suppressed the activation of the PI3K/Akt pathway. (a) Western blot analysis of p-PI3K/PI3K and p-Akt/Akt. (b) The relative protein content was quantified by densitometry. Results were performed at least 3 times. Data are shown as the means ± standard error of the mean. ^∗^*p* < 0.05 vs. the control group. ^#^*p* < 0.05 versus the Rb1 20 *μ*g/mL group.

**Figure 8:**
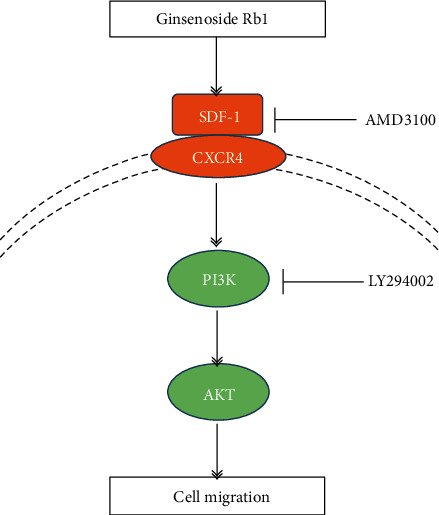
Working model of ginsenoside Rb1-mediated BMSC migration. Ginsenoside Rb1 improved the migration of BMSCs through the SDF-1/CXCR4 axis and PI3K/Akt pathway, and this stimulus could be suppressed by AMD3100 and LY294002.

CXCR4 is a specific membrane receptor for SDF-1, and the SDF-1/CXCR4 axis is widely investigated to evaluate the migration ability in various species. Increasing evidence indicated that the decreased cell migration property may be related to the downregulation of CXCR4 [26–28]. In addition, Xu et al. [29] reported that PAX3 may facilitate the migration of neural crest cells by increasing the CXCR4 expression. To the best of our knowledge, there have been few studies on the effect of ginsenoside Rb1 on the SDF-1/CXCR4 axis. Based on real-time PCR and Western blot analysis, we found that the treatment of ginsenoside Rb1 increased both the gene and protein expression of CXCR4 and the PI3K/Akt pathway in BMSCs (Figure 3). BMSCs were preincubated with AMD3100 (CXCR4 antagonist), resulting in significant suppression of the ginsenoside Rb1-mediated upregulation of the migration index (Figure 4), which was matched with the downregulation of CXCR4 expression (Figure 5). In addition, the pretreatment of LY294002 (PI3K inhibitor) suppressed the cell migration of BMSCs (Figure 6), accompanied by the decreased expression of p-PI3K/PI3K and p-Akt/Akt (Figure 7), indicating that the positive influences of ginsenoside Rb1 on cell migration may be related to the activation of the SDF-1/CXCR4 axis and PI3K/Akt pathway. Moreover, our results showed that the blockage of the SDF-1/CXCR4 axis induced the downregulated phosphorylation of PI3K and Akt, indicating that the PI3K/Akt pathway may participate in the function of the SDF-1/CXCR4 axis during BMSC migration (Figure 5). Therefore, we proposed that ginsenoside Rb1 may promote the migration of BMSCs through activation of the SDF-1/CXCR4 axis and PI3K/Akt pathway (Figure 8). Further studies to evaluate the effect of ginsenoside Rb1 on the migration of BMSCs in a diabetes-induced wound animal model may be worthwhile in understanding the contribution of ginsenoside Rb1 in the wound-healing process.

Taken together, this study demonstrated the effect and underlying mechanism of ginsenoside Rb1 on the migration of BMSCs, and these findings may be helpful to identify new drugs to promote wound healing.

## Data Availability

Access to data is restricted; the raw/processed data required to reproduce these findings cannot be shared at this time as the data also forms part of an ongoing study.
